# Integrated characterization of six sesquiterpene synthases unravels diversified terpene biosynthesis in the mint species *Leucosceptrum canum*

**DOI:** 10.3389/fpls.2026.1833733

**Published:** 2026-04-28

**Authors:** Qinqin Shen, Shaojun Cheng, Chenxiao Zhao, Zonghua Xiao, Yan Liu, Shenghong Li

**Affiliations:** 1 State Key Laboratory of Southwestern Chinese Medicine Resources, and Innovative Institute of Chinese Medicine and Pharmacy, Chengdu University of Traditional Chinese Medicine, Chengdu, China; 2 State Key Laboratory of Phytochemistry and Natural Medicines, and Yunnan Key Laboratory of Natural Medicinal Chemistry, Kunming Institute of Botany, Chinese Academy of Sciences, Kunming, China

**Keywords:** cyclization mechanism, germacrene A, *Leucosceptrum canum*, sesquiterpene synthases, metabolic engineering, *α-trans-bergamotene*

## Abstract

**Introduction:**

Sesquiterpenoids are important plant-derived compounds with diverse bioactivities and significant pharmaceutical potential. *Leucosceptrum canum* Smith, a monotypic species of the Lamiaceae family, accumulates a diverse array of terpenoids with important biological functions. However, the functional diversity and catalytic mechanisms of its sesquiterpene synthases (sesquiTPSs) remain poorly understood.

**Methods:**

Six sesquiTPSs (LcTPS3-LcTPS8) from *L. canum* were functionally characterized through heterologous expression in *Escherichia coli*, followed by product identification using GC-MS and NMR analyses. Phylogenetic analysis, molecular docking, and gene expression profiling were performed to investigate evolutionary relationships, catalytic mechanisms, and expression patterns. Metabolic engineering strategies were applied to construct high-yield production systems.

**Results:**

LcTPS3-LcTPS8 generated germacrene A, (+)-5-epi-aristolochene, γ-selinene, germacrene D, α-trans-bergamotene, and (+)-β-himachalene, respectively. Phylogenetic analysis clustered LcTPS3-6 and LcTPS8 in the TPS-a subfamily and LcTPS7 in TPS-b, reflecting their evolutionary divergence. Integrated structural and docking analyses revealed four distinct cyclization modes, with the spatial conformation of FPP determining product specificity. Expression analysis demonstrated tissue-specific patterns and stress responses, suggesting a regulated metabolic network. We established production systems for six sesquiterpenoids, with titers reaching up to 618.73 mg L⁻¹ in shake-flask cultures.

**Discussion:**

These findings provide mechanistic insights into sesquiTPS function and terpene diversification in *L. canum*, and offer valuable enzymatic resources for the sustainable biosynthesis of high-value sesquiterpenoids.

## 1 Introduction

**Figure 1:**
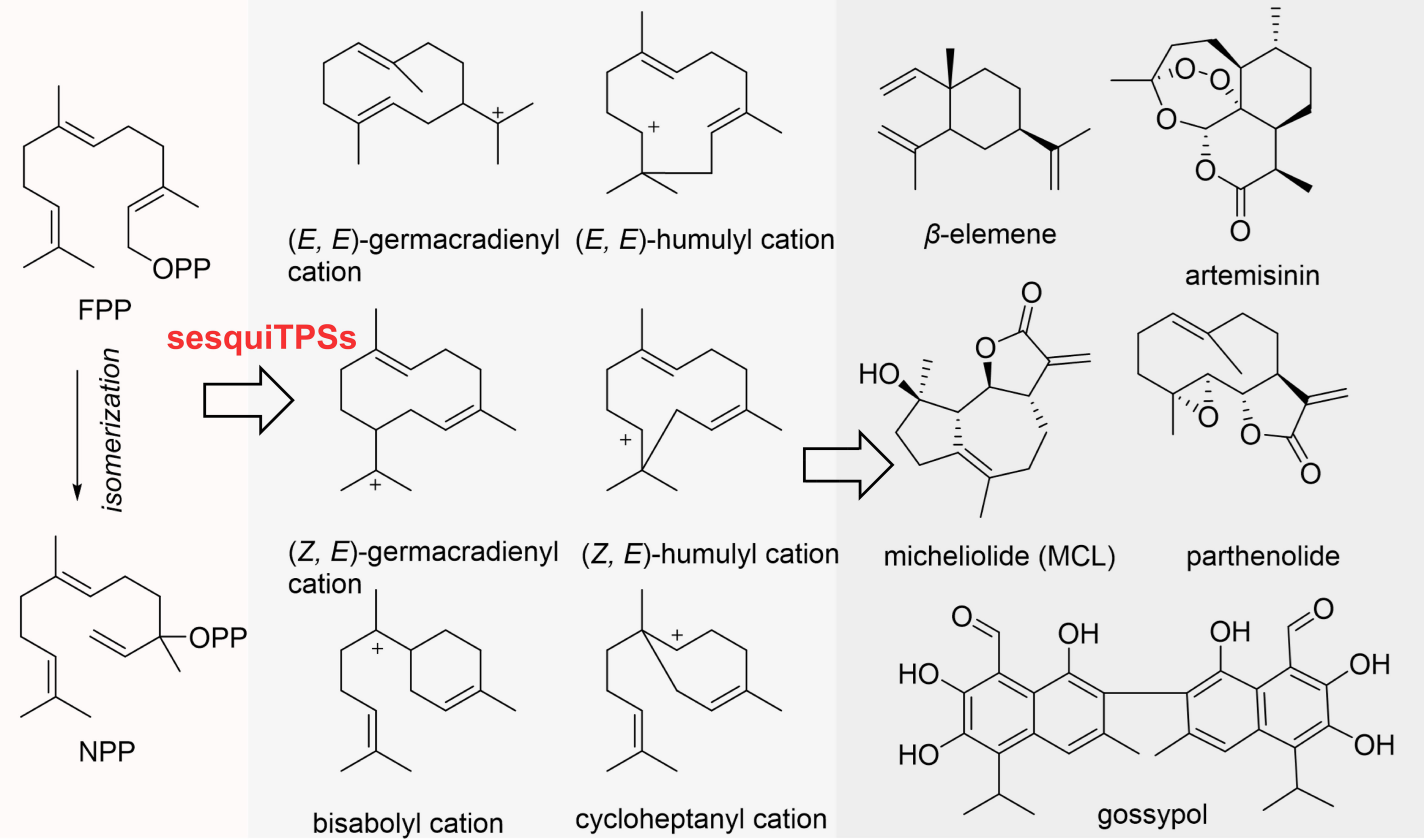
The representative plant-derived sesquiterpenoids and their biosynthetic pathway.

Sesquiterpenoids, the largest and most structurally diverse class of terpenoids, represent a vast superfamily encompassing over 100,000 natural products (Fraga, 2012; Chen et al., 2020; Wei et al., 2025). Composed of three isoprene units, these compounds exhibit a wide range of scaffolds, including acyclic, monocyclic, bicyclic, and polycyclic structures, and possess broad biological activities with significant potential for application (Degenhardt et al., 2009; Christianson, 2017; Durairaj et al., 2019). Numerous sesquiterpenoids have been developed into highly valuable medicinal molecules or key agents for plant stress resistance. As illustrated in **Figure 1**, artemisinin, extracted from *Artemisia annua* L., serves as a first-line therapeutic agent against malaria, significantly reducing the global incidence and mortality associated with this disease (Chen et al., 2025; Wang et al., 2020). Gossypol, isolated from cottonseeds, not only acts as a natural phytoalexin for plants but also exhibits notable antitumor activity, making it a valuable lead compound for drug development (Cao et al., 2021; Renner et al., 2022). *β*-elemene, an active component derived from the traditional medicinal plant *Curcuma wenyujin* Y.H.Chen & C.Ling is classified as a National Class II non-cytotoxic antitumor drug in China and has been extensively utilized clinically for cancer treatment (Jiang et al., 2017; Zhai et al., 2018). Parthenolide exerts its antitumor effects by inducing apoptosis of cancer stem cells and inhibiting the proliferation of cancer cells (Karam et al., 2021; Xu et al., 2025). Micheliolide (MCL), a valued natural product, is a promising candidate for anticancer drug development, with its derivative ACT001 having been successfully applied in clinical trials for the treatment of malignant glioma (Karam et al., 2021; Ren et al., 2025). These structurally diverse and functionally versatile sesquiterpenoids play irreplaceable roles in the pharmaceutical and agricultural sectors, serving as a privileged natural library for novel drug and pesticide discovery.

Sesquiterpenoid biosynthetic pathways exhibit a highly conserved initiation module, with all derivatives originating from the universal precursor farnesyl pyrophosphate (FPP) (Christianson, 2017). Nerolidyl diphosphate (NPP) is synthesized through the isomerization of FPP (**Figure 1**). At the active site of sesquiTPSs, the pyrophosphate moiety (PPi) of FPP undergoes ionization with the assistance of Mg^2+^. This step serves as a critical trigger for the subsequent rearrangement of the carbon skeleton. Following ionization, the resulting carbocations engage in 1,6-, 1,7-, 1,10-, or 1,11-cyclization to produce a series of characteristic monocyclic carbocation intermediates (Degenhardt et al., 2009; Hong and Tantillo, 2014; Christianson, 2017; Durairaj et al., 2019), including (*E*, *E*)-germacradienyl cation, (*E*, *E*)-humulyl cation, (*Z*, *E*)-germacradienyl cation, (*Z*, *E*)-humulyl cation, bisabolyl cation, and cycloheptanyl cation (**Figure 1**). These labile carbocation intermediates undergo a cascade of catalytic rearrangement events, including intramolecular proton transfer, specific cyclization, hydride migration, and methyl migration (Tantillo, 2017; Dickschat, 2019). Ultimately, they experience deprotonation or functional group incorporation via nucleophilic addition, resulting in the formation of structurally diverse sesquiterpenes.

*Leucosceptrum canum* Smith*.*, a monotypic species belonging to the Lamiaceae family, is a large woody plant distributed from the Himalayas to southwestern China. In traditional Chinese medicine, its leaves are clinically employed for the management of inflammatory conditions, such as hepatitis, gastritis, cutaneous ulcers, and pustular dermatitis (Guo et al., 2024). A large number of sesterterpenoids with a unique 5/6/5 core structure, diterpenoids, and sesquiterpenoids have been isolated from *L. canum* (Luo et al., 2010; Luo et al., 2012; Luo et al., 2013; Luo et al., 2011; Guo et al., 2019; Zhang et al., 2021; Guo et al., 2021, 2024). Through mining the transcriptome of *L. canum* glandular trichomes, three key enzymes have been functionally characterized, including a geranylfarnesyl diphosphate synthase (GFDPS), synthesizing the universal C_25_ sesterterpenoid precursor (Liu et al., 2016), a TPS-a enzyme LcTPS2 capable of producing macrocyclic diterpenes and sesterterpenes (Chen et al., 2021), and a sesquiTPS (Lc-Ceds) that produces cedrol (Luo et al., 2019). Diverse terpenoids accumulated in *L. canum* may underpin its chemical defense strategies, making the functional characterization of terpene synthases crucial for elucidating terpenoid diversification and ecological adaptation.

In this study, we functionally characterized six sesquiTPSs (LcTPS3-LcTPS8) from the full-length transcriptome data of *L. canum*. Through heterologous expression and product identification, we defined their catalytic functions. Based on canonical sesquiterpene biosynthesis, molecular docking revealed how FPP conformation in the active sites of LcTPSs determines their distinct cyclization pathways. In addition, qRT-PCR analysis revealed tissue-specific expression patterns and stress responsiveness of these genes. Overall, these findings provide valuable catalytic tools for the synthetic biology-based production of target sesquiterpenoids and deepen our understanding of the catalytic specificity of sesquiTPSs.

## 2 Materials and methods

### 2.1 Plant materials

*Leucosceptrum canum* Smith was obtained from Kunming Botanical Garden (Yunnan Province, China) and maintained in a glasshouse (24 °C, 16 hours light/8-hour dark cycle). The collected plant materials included leaves (L), roots (R), stem epidermis (SE), epidermis-free stems (S), and trichomes (T). Stress treatments comprised: hydroponic culture with 0.5 mM CuCl_2_, foliar spraying with 0.5 mM MeJA or 1 mM SA, and UV irradiation for 20 minutes. Leaf samples were harvested 48 hours post-treatment and stored at -70 °C for subsequent experiments.

### 2.2 Gene cloning and plasmid construction

Candidate sesquiterpene synthases (sesquiTPSs) were identified by tBLASTn search against the *L. canum* full-length transcriptome using amino acid sequences of known plant sesquiterpenes. Total RNA was extracted from *L. canum* leaves using the TRIzol Plus RNA Purification Kit (Sangon Biotech), and cDNA was synthesized using the HiScript III First Strand cDNA Synthesis Kit (Vazyme Biotech). The full-length coding sequences of *LcTPS3-LcTPS8* were amplified by PCR using *Phanta*^®^ Max SuperFidelity DNA polymerase (Vazyme, P515). Amplified fragments were subcloned into *BamH* I and *Xho* I restriction sites of the pET32a vector via homologous recombination by 2×ClonExpress Mix (Vazyme C115), and then transferred into *E. coli* (DH5α) using a heat shock method. The positive colonies were identified using PCR and then subjected to Sanger sequencing, with resultant plasmids designated pET32a-LcTPS3 to pET32a-LcTPS8. The plasmids constructed in this study are shown in **Supplementary Table S3**. All primers used are listed in **Supplementary Table S4**.

### 2.3 Sequence alignment and phylogenetic analysis

The protein sequences of sesquiTPSs and other terpene synthases (TPSs) were retrieved from the NCBI database (**Supplementary Table S2**). Sequence alignments were performed using CLC Bio Sequence Viewer 7, and phylogenetic trees were constructed by the maximum likelihood method with bootstrap support (1,000 replicates) indicated at nodes. The presented phylogenetic trees were plotted using ChiPlot (Xie et al., 2023).

### 2.4 Functional characterization of LcTPSs in *Escherichia coli*

The recombinant plasmid pET32a-LcTPS3 to pET32a-LcTPS8 and the pET28a-MmGFDPS (Geranylfarnesyl diphosphate synthase from the archaeon *Methanosarcina mazei*) plasmid were co-transformed into the *E. coli* expression strain C41(DE3), which harbors the pBbA5c vector encoding the complete MVA pathway and FPP synthase genes (Li et al., 2021). The empty pET32a vector was transformed as a negative control. The colonies were selected on the LB agarose plate containing 34 μg mL^-1^ chloramphenicol and 100 μg mL^-1^ ampicillin, confirmed by colony-PCR. The confirmed transformants were inoculated in 50 mL TB liquid medium (24 g L^-1^ yeast extract, 12 g L^-1^ tryptone, 10 mL glycerol, 2.31 g L^-1^ KH_2_PO_4_, and 12.54 g L^-1^ K_2_HPO_4_) containing the corresponding antibiotics in 250 mL flasks at 37 °C, 200 rpm min^-1^ to an OD_600_ of 0.8, and then induced by 1 mM IPTG at 16 °C for 16 h, followed by continuing cultivation at 25 °C for 3 days. Subsequently, the culture media were extracted with 50 mL of *n*-hexane, and the obtained organic phase was condensed to 1 mL for GC-MS analysis.

GC-MS analysis was performed in an Agilent 8890 GC/7000D MS system equipped with an HP-5MS column (28 m × 0.25 mm × 0.25 μm). Helium was used as carrier gas at 1.2 mL min^−1^ in splitless mode (1 μL injection, injector temperature 250 °C). The GC oven was programmed as follow*s*: initial temperature 60 °C (hold 1 min), ramp at 40 °C min^-1^ to 180 °C (hold 1 min), then 25 °C min^-1^ to 270 °C, finally 20 °C min^-1^ to 300 °C (hold 2 min). MS detection was performed in EI mode at 70 eV (ion source 230 °C, transfer line 280 °C) with m/z 50–450 scanning after a 4 min solvent delay. Mass spectral data were matched against the NIST 10 database for prediction.

### 2.5 Isolation and structural characterization of compounds produced by LcTPSs

Five recombinant strains harboring pET32a-LcTPS3, pET32a-LcTPS4, pET32a-LcTPS5, pET32a-LcTPS6, and pET32a-LcTPS8, respectively, were constructed in an engineered *E. coli* chassis containing the pBbA5c vector for enhanced FPP supply, and were fermented in 5 L TB medium under the conditions described previously. The culture was extracted twice using ethyl acetate (EtOAc) and then evaporated under reduced pressure. The crude extract was subjected to silica gel column chromatography and eluted with n-hexane. The n-hexane fraction was further purified by chromatography on an alumina column, followed by a silver nitrate-silica gel (1:9, w/w) column using n-hexane as the eluent, affording compounds **1** (650 mg), **2** (730 mg), **3** (61 mg), **4** (15 mg), and **6** (21 mg). The structures were determined by NMR experiments on a 600MHz Bruker, with complete spectral data available in **Supplementary Figures S3–S12**.

^1^H and ^13^C NMR data of germacrene A (1) (Zhao et al., 2021)

^1^H NMR (600 MHz, C_6_D_6_) *δ*_H_: 4.79 (s, 1H), 4.78 – 4.74 (m, 1H), 4.68 (s, 1H), 4.52 (d, *J* = 10.7 Hz, 1H), 1.78– 2.37 (m, 9H), 1.70 (m, 1H), 1.67 (s, 3H), 1.50 (m, 1H), 1.42 (s, 3H), 1.32 (s, 3H). ^13^C NMR (150 MHz, C_6_D_6_) *δ*_C_:153.6 (C), 137.9 (C), 132.0 (CH), 128.8 (C), 126.9 (CH), 107.9 (CH_2_), 51.8 (CH), 42.0 (CH_2_), 39.9 (CH_2_), 35.3 (CH_2_), 34.1 (CH_2_), 27.1 (CH_2_), 20.4 (CH_3_), 16.8 (CH_3_), 16.3 (CH_3_).

^1^H and ^13^C NMR data of (+)-5-*epi*-aristolochene (2) (Whitehead et al., 1990)

^1^H NMR (600 MHz, CDCl_3_) *δ*_H_: 5.54 (dt, *J* = 6.6, 2.0 Hz, 1H), 4.70 (s, 1H), 4.68 (s, 1H), 2.30 – 2.21 (m, 2H), 2.04 – 1.94 (m, 3H), 1.81 (dddd, *J* = 15.8, 13.3, 4.1, 2.0 Hz 1H), 1.74 (s, 3H), 1.69 (dt, *J* = 13.9, 3.0 Hz, 1H), 1.57 – 1.46 (m, 3H), 1.29 (t, *J* = 13.5 Hz, 2H), 1.18 (s, 3H), 1.00 (d, *J* = 7.1 Hz, 3H). ^13^C NMR (151 MHz, CDCl_3_) *δ*_C_:150.5 (C), 141.3 (C), 120.9 (CH), 108.3 (CH_2_), 44.1 (CH_2_), 41.7 (CH), 40.1 (CH), 39.0 (C), 32.2 (CH_2_), 30.4 (CH_2_), 30.3 (CH_3_), 30.2 (CH_2_), 22.6 (CH_2_), 21.2 (CH_3_), 17.6 (CH_3_).

^1^H and ^13^C NMR data of *γ*-selinene (3) (Zhao et al., 2021)

^1^H NMR (600 MHz, CDCl_3_) *δ*_H_: 4.72 (s, 1H), 4.71 (s, 1H), 2.55 (dt, *J* = 13.0, 2.5 Hz, 1H), 2.04 – 1.96 (m, 1H), 1.91 – 1.78 (m, 3H), 1.76 (s, 3H), 1.61 (s, 3H), 1.60 – 1.47 (m, 6H), 1.34 – 1.24 (m, 2H), 1.05 (s, 3H). ^13^C NMR (150 MHz, CDCl_3_) *δ*_C_: 151.0 (C), 135.0 (C), 124.7 (C), 108.2 (CH_2_), 47.0 (CH), 42.4 (CH_2_), 40.4 (CH_2_), 34.6 (C), 33.3 (CH_2_), 30.9 (CH_2_), 27.8 (CH_2_), 24.8 (CH_3_), 21.0 (CH_3_), 19.4 (CH_3_), 19.2 (CH_2_).

^1^H and ^13^C NMR data of germacrene D (4) (Randriamiharisoa et al., 1986)

1H NMR (600 MHz, CDCl_3_) *δ*_H_: 5.78 (d, *J* = 15.9 Hz, 1H), 5.25 (dd, *J* = 15.9, 9.9 Hz, 1H), 5.13(dd, *J* = 11.2, 4.8 Hz, 1H), 4.79 (d, *J* = 2.4 Hz, 1H), 4.74 (d, *J* = 2.4 Hz, 1H), 2.44 – 1.98 (m, 7H), 1.51 (s, 3H), 1.48 (m, 1H), 1.42 (m, 1H), 0.87 (s, 3H), 0.81 (d, *J* = 6.8 Hz, 3H), 0.79 (m, 1H). ^13^C NMR (150 MHz, CDCl_3_) *δ*_C_:149.0 (C), 135.6 (CH), 134.2 (C), 133.7 (CH), 129.8 (CH), 109.2 (CH_2_), 53.1 (CH), 40.9 (CH_2_), 34.6 (CH_2_), 32.9 (CH), 29.4 (CH_2_), 26.7 (CH_2_), 20.9 (CH_3_), 19.5 (CH_3_), 16.1 (CH_3_).

^1^H and ^13^C NMR data of (+)-*β*-himachalene (6) (Ho and Chein, 2006)

^1^H NMR (600 MHz, CDCl_3_) *δ*_H_: 5.42 (s, 1H), 2.89 (s, 1H), 2.63 (m, 1H), 2.45 (m, 1H), 1.97 – 1.86 (m, 4H), 1.74 (s, 3H), 1.71 (s, 3H), 1.47 – 1.38 (m, 4H), 0.97 (s, 3H), 0.73 (s, 3H). ^13^C NMR (150 MHz, CDCl_3_) *δ*_C_:134.9 (C), 131.3 (C), 129.2 (CH), 122.6 (CH), 46.3 (CH), 45.3 (CH_2_), 34.7 (C), 34.2 (CH_2_), 30.4 (CH_2_), 29.4 (CH_3_), 26.2 (CH_2_), 24.3 (CH_3_), 23.8 (CH_3_), 21.6 (CH_2_), 20.4 (CH_3_).

### 2.6 Expression and purification of recombinant proteins

The recombinant plasmids encoding six terpene synthases were individually transformed into *Escherichia coli* BL21(DE3). Positive clones were cultured in LB medium supplemented with 100 μg mL^-1^ ampicillin at 37 °C with shaking at 200 rpm until OD_600_ reached 0.6. Protein expression was induced with 0.5 mM IPTG, followed by incubation at 16 °C and 200 rpm for 16 h. Cells were harvested by centrifugation at 8,000 rpm for 5 min at 4 °C and resuspended in lysis buffer A (50 mM Tris, pH 7.5, 300 mM NaCl, 10% glycerol). Cell disruption was performed using a low-temperature ultra-high-pressure cell disruptor (JNBIO^®^), and the lysate was collected by centrifugation at 12,000 rpm for 1 h at 4 °C. The soluble fraction was applied to an ÄKTA pure system equipped with a HisTrap FF column for affinity purification. Bound proteins were eluted with buffer B (50 mM Tris-HCl, pH 7.5, 300 mM NaCl, 250 mM imidazole, 5 mM DTT, 10% glycerol). Eluted proteins were concentrated using an Amicon Ultra-15 centrifugal filter unit (Millipore) and buffer-exchanged into 50 mM Tris-HCl (pH 7.5) containing 10% glycerol. Protein concentration was determined by absorbance at 280 nm using theoretical extinction coefficients and was used immediately for enzymatic assays. The purified proteins were analyzed by SDS-PAGE.

### 2.7 *In vitro* enzyme activity

For product profiling assays, *in vitro* enzyme reactions were carried out in a total volume of 200 μL containing 50 mM Tris–HCl (pH 7.5), 5 mM MgCl_2_, 5% (*v/v*) glycerol, and 2 mM DTT. Reactions contained 200 μg of purified recombinant LcTPS protein and 50 μM substrate (GPP; FPP or GGPP), and were incubated at 30 °C for 1 h. Products were extracted into 150 μL of n-hexane, and 100 μL of the supernatant was transferred to a sample vial for GC–MS analysis with a 4 μL injection volume.

### 2.8 Kinetic assays

Initial assays were conducted to verify enzymatic activity and to determine the linear range of the reaction with respect to incubation time and enzyme concentration. Based on these preliminary experiments, enzyme amounts and reaction times were individually optimized for each LcTPS variant to ensure that measurements were performed under initial rate conditions. Enzyme kinetic assays were carried out at 30 °C in a total volume of 200 μL containing 50 mM Tris–HCl (pH 7.5), 5 mM MgCl_2_, 5% (*v/v*) glycerol, and 2 mM DTT. FPP was supplied at seven concentrations, ranging from 50 μM to 0.78 μM in a two-fold serial dilution. The optimized enzyme amounts and incubation times were as follows: LcTPS3 (1 μg, 10 min), LcTPS4 (1 μg, 10 min), LcTPS5 (2 μg, 10 min), LcTPS6 (5 μg, 10 min), LcTPS7 (1 μg, 5 min), and LcTPS8 (5 μg, 10 min). Reactions were initiated by the addition of the enzyme and terminated by snap-freezing in liquid nitrogen. Product extraction and GC–MS analysis were performed as described for the *in vitro* enzyme activity assays. Quantification was performed using external standard curves.

### 2.9 Molecular docking

The protein structures of LcTPSs were predicted using AlphaFold3 (Callaway, 2024), and the highest-ranked predicted structure was selected for further modeling. Molecular docking of LcTPSs with their substrate FPP was performed using AutoDock Vina. The substrate structure was first energy-minimized, and then the docking grid box (22 × 22 × 22 Å^3^) was centered on the conserved active pocket characteristic of sesqiuTPSs. Docking simulations used the Lamarckian genetic algorithm, and multiple independent computations were performed. The generated conformations were grouped by binding free energy values, and the conformation with the lowest binding free energy (representing the most stable state) was finally chosen as the typical model to analyze the interaction sites between the substrate and *LcTPSs*. Visualization employed PyMOL for 3D structures and LigPlot+ for 2D diagrams.

### 2.10 Gene expression analysis

Real-time qRT-PCR was performed using the BIO-RAD CFX instrument and ArtiCanATM SYBR reagent (Tsingke Biotech). The *actin* gene of *L. canum* was used as an internal control, and relative expression levels were calculated using the 2^-ΔΔCt^ method. Each sample had three biological replicates.

### 2.11 Shake-flask fermentation

Six engineered *E. coli* strains were constructed by introducing the LcTPSs (pET32a-LcTPS3 to pET32a-LcTPS8) into a chassis harboring the pBbA5c vector, which contains seven genes of the MVA pathway and an FPP synthase gene to enhance FPP supply (Peralta-Yahya et al., 2011). pET28a-MmGFDPS (Ogawa et al., 2010) was not introduced, as its presence would redirect the metabolic flux toward GGPP and GFPP.

For each strain, 20 mL fermentations were carried out in 250 mL flasks following the aforementioned conditions. After induction at 16 °C, sodium pyruvate was supplemented to a final concentration of 20 mM, along with 2% (*v/v*) glycerol as an additional carbon source. After fermentation, 20 mL of *n*-hexane was added to the culture, followed by incubation at 25 °C with shaking at 200 rpm for 30 min. A 1 mL aliquot of the organic phase was collected for GC–MS analysis, and product yields were quantified based on external standard calibration curves. Standard curves were prepared using the isolated compounds, yielding six curves: germacrene A (y = 755605x – 290056, R² = 0.9996), *α*-*trans*-bergamotene (y = 614072.4x + 15718, R² = 0.9998), (+)-*β*-himachalene (y = 193849x + 54711, R² = 0.9997), germacrene D (y = 541800x - 8019.1, R² = 0.9999), *γ*-selinene (y = 711448x + 317520, R² = 0.9998), and (+)-5-*epi*-aristolochene (y = 867660x – 278241, R² = 0.9991).

### 2.12 Accession numbers

The nucleotide sequences of LcTPS3–8 have been submitted to NCBI under the following accession numbers: PX766225, PX766226, PX766227, PX766228, PX766229, PX766230.

## 3 Results

### 3.1 Identification of LcTPSs from *Leucosceptrum canum*

To identify sesquiTPSs in *Leucosceptrum canum*, a tBLASTn search was conducted against its full-length transcriptome using functionally characterized sesquiTPSs as query sequences. Candidate TPS genes were further screened based on the presence of full-length open reading frames and conserved TPS motifs. Phylogenetic analysis was performed to identify TPS candidates from sesquiterpene-related subfamilies. In addition, genes showing relatively higher expression in leaves based on qRT-PCR analysis were prioritized for further functional characterization. Based on these criteria, six candidate TPS genes (*LcTPS3*–*LcTPS8*) were cloned from leaf cDNA, and their sequence lengths and Swiss-Prot annotations are summarized in **Supplementary Table S1**.

**Figure 2:**
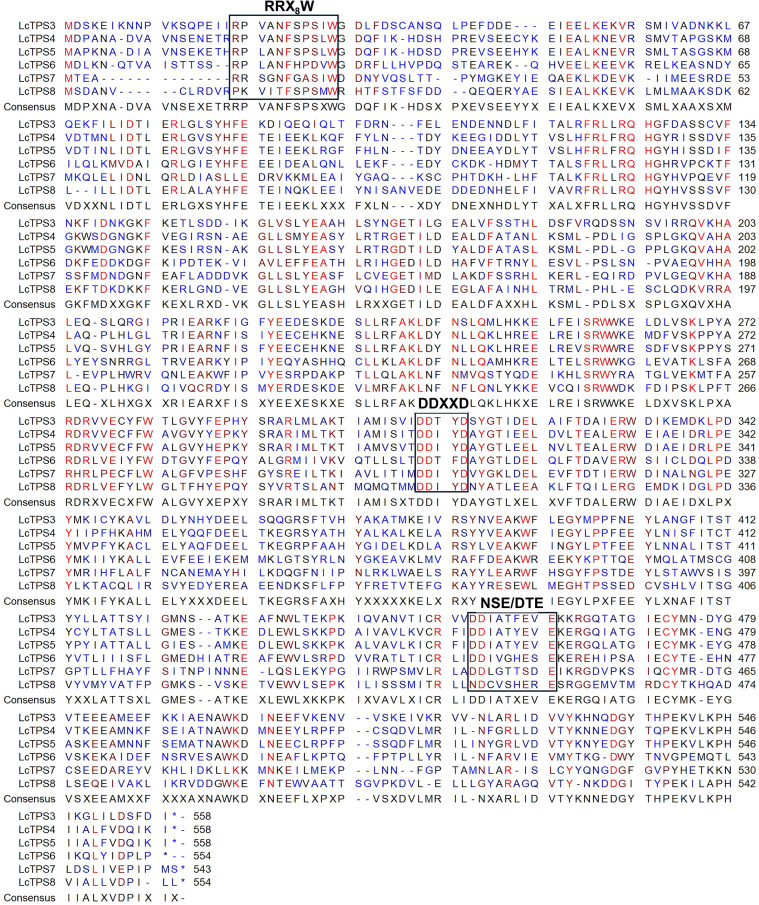
Multiple sequence alignment of LcTPSs. Conserved functional domains RRX_8_W, DDXXD, and NSE/DTE are highlighted in black boxes.

**Figure 3:**
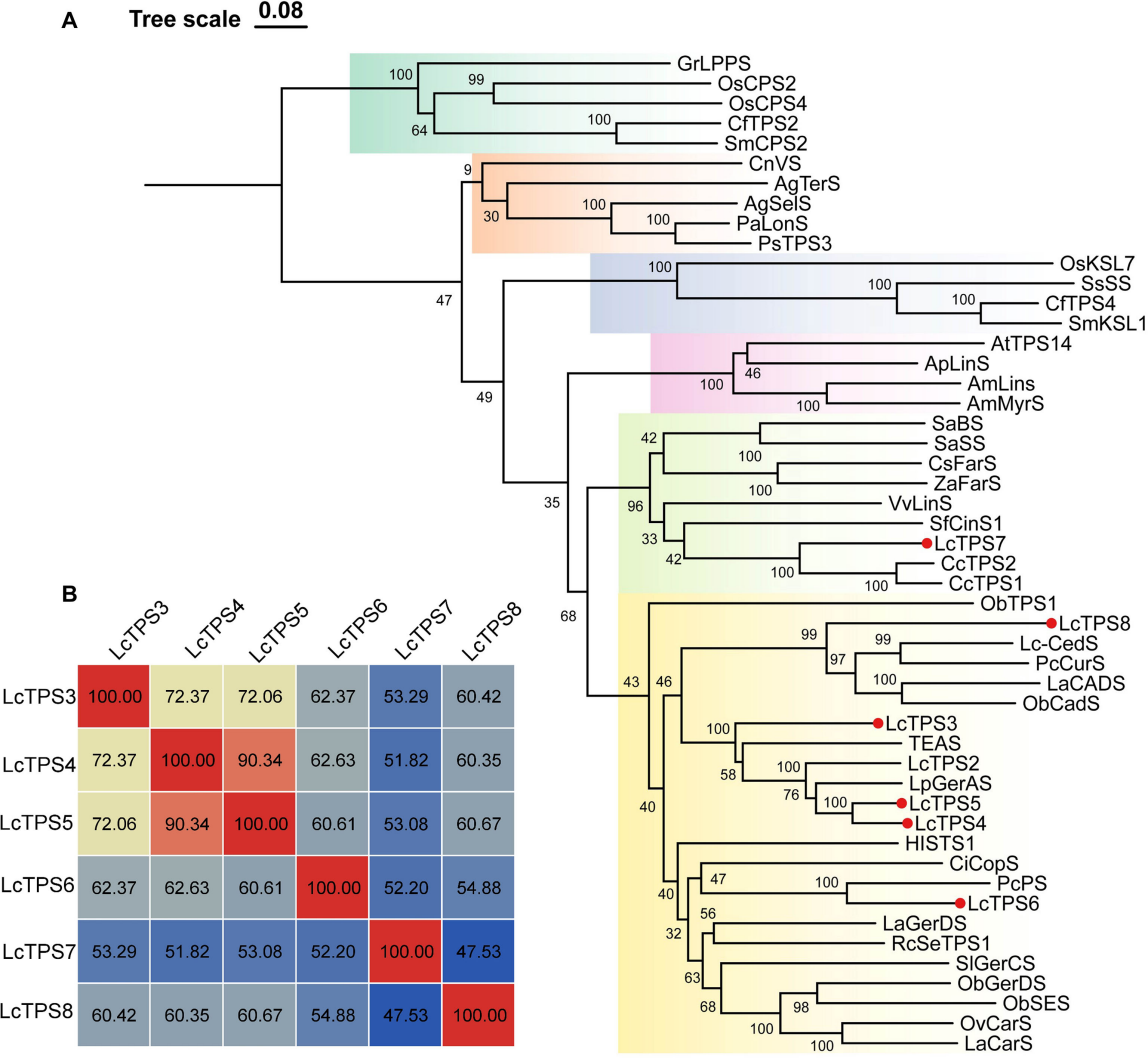
Phylogenetic and sequence similarity analysis of LcTPSs. **(A)** The tree was constructed by the maximum likelihood method. The TPS subfamilies a, b, c, d, e/f, and g are shaded yellow, green, cyan, brown, blue, and purple, respectively. The candidate genes identified in this study are marked with red circles. **(B)** Amino acid sequence similarity analysis. The numbers in the blocks indicate the sequence similarity values.

Multiple sequence alignment confirmed that six LcTPSs are classified as Class I TPSs, which are defined by the conserved N-terminal RR(X)_8_W motif and C-terminal motifs (DDXXD and NSE/DTE) (**Figure 2**) (Christianson, 2017). LcTPS4 and LcTPS5 exhibited a sequence similarity of 90.34% (**Figure 3B**), while the similarity among the remaining genes was below 75%, indicating potential catalytic functional divergence. The TPS-a and TPS-b subfamilies are predominantly composed of monoterpene and sesquiterpene synthases from angiosperms, and phylogenetic analysis demonstrated that LcTPS3, LcTPS4, LcTPS5, LcTPS6, and LcTPS8 are members of the TPS-a subfamily (**Figure 3A**). LcTPS8 clustered in the same phylogenetic clade as (*E*)-*β*-caryophyllene synthase (ObTPS1), suggesting a potential initial 1,11-cyclization. LcTPS3–LcTPS6 were predicted to undergo initial 1,10-cyclization, because LcTPS3, 4, and 5 clustered with 5-*epi*-aristolochene synthase (TEAS) and germacrene A synthase (LpGerAS), while LcTPS6 grouped with germacrene D synthase (LaGerDS) and patchoulol synthase (PcPS). Additionally, LcTPS7 belongs to the TPS-b subfamily (**Figure 3A**), clustering with the previously reported promiscuous terpenoid synthase (CcTPS1) and (*R*)-*β*-bisabolene synthase (CcTPS2) from *Colquhounia coccinea* var. *mollis*, and is anticipated to mediate 1,6-cyclization.

### 3.2 Functional characterization of LcTPSs in *Escherichia coli*

**Figure 4:**
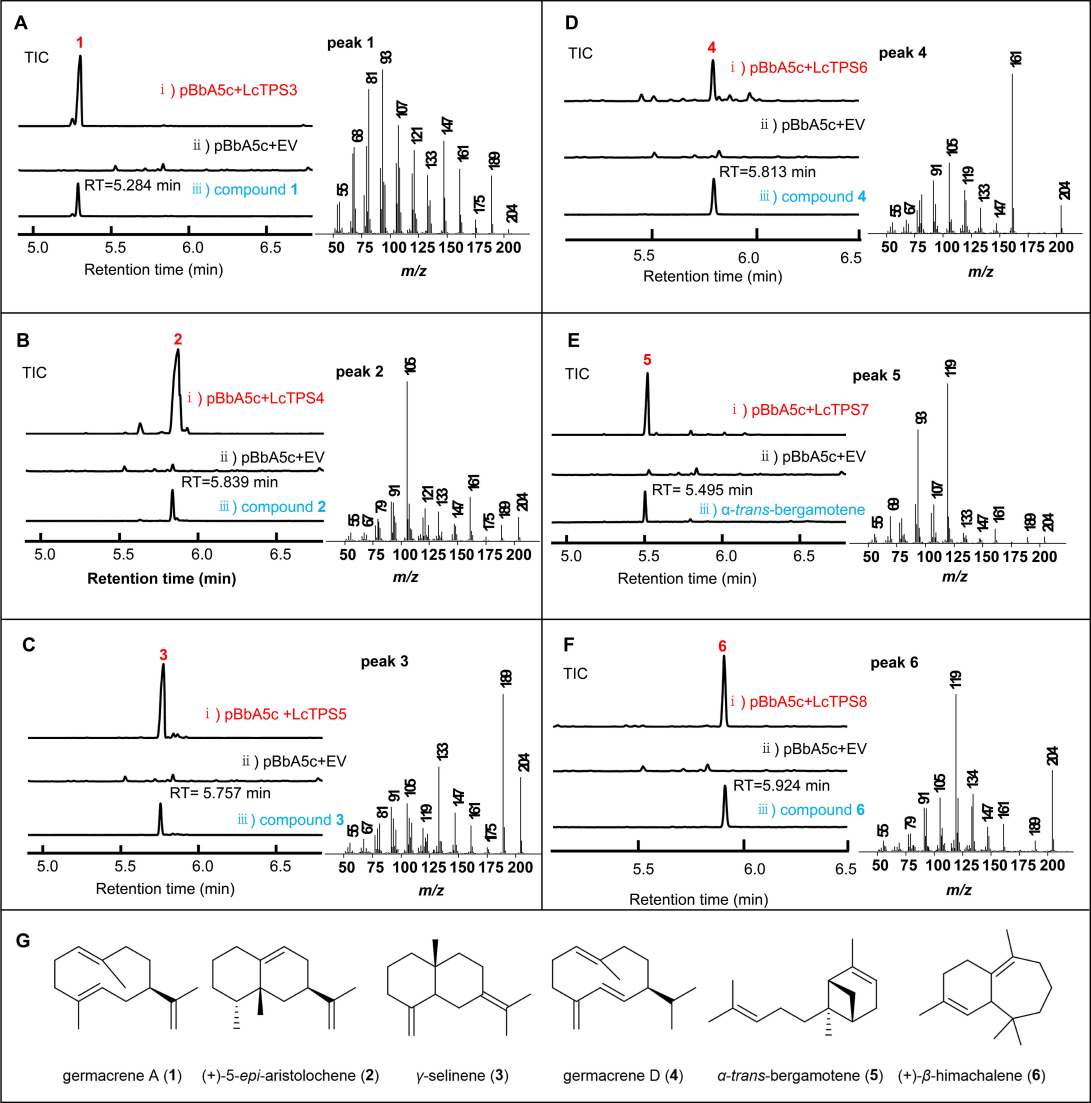
Functional characterization of LcTPSs in *E. coli.* **(A–F)** GC-MS analysis of the extracts of the *E. coli* strains expressing LcTPS3, LcTPS4, LcTPS5, LcTPS6, LcTPS7, and LcTPS8, respectively, along with the mass spectra of products. Co-expression of pBbA5c and empty vector (EV) was used as the negative control. **(G)** Chemical structures of the products generated by LcTPS3–LcTPS8.

Six candidate sesquiTPSs were heterologously expressed in engineered *Escherichia coli* strains developed in our laboratory (Li et al., 2021). These strains were constructed by coexpressing a promiscuous GFDPS from *Methanosarcina mazei* (MmGFDPS) (Ogawa et al., 2010), a heterologous mevalonate (MVA) pathway comprising seven genes, and an additional FPP synthase gene (Peralta-Yahya et al., 2011), thereby providing diverse polyprenyl diphosphate precursors, including GPP, FPP, GGPP, and GFPP, and ensuring their sufficient supply. Under this system, all six enzymes utilized FPP as the substrate and produced six distinct terpenoid compounds. GC-MS analysis indicated that LcTPS3 generated a specific product with a retention time of 5.284 min, which was predicted as *β*-elemene through NIST10 mass spectral library matching (**Figure 4A**). Similarly, LcTPS4–8 generated compounds with retention times of 5.839 min (**2**), 5.757 min (**3**), 5.813 min (**4**), 5.495 min (**5**), and 5.924 min (**6**), respectively (**Figure 4**).

The identity of compound 5 was confirmed as *α*-*trans*-bergamotene by comparison with an authentic standard, showing identical retention time and mass spectrum (**Figure 4E**), indicating that LcTPS7 functions as an *α*-*trans*-bergamotene synthase. To determine the structures of the remaining products, large-scale (5 L) fermentation was performed for strains expressing LcTPS3, LcTPS4, LcTPS5, LcTPS6, and LcTPS8. The products were extracted, purified by silica gel chromatography, and structurally characterized by NMR spectroscopy, leading to their identification as germacrene A (**1**) (Zhao et al., 2021), (+)-5-*epi*-aristolochene (**2**) (Whitehead et al., 1990), *γ*-selinene (**3**) (Zhao et al., 2021), germacrene D (**4**) (Randriamiharisoa et al., 1986), (+)-*β*-himachalene (**6**) (Ho and Chein, 2006) via NMR spectroscopy (**Figure 4G**).

Notably, although LcTPS3 was initially assigned as producing *β*-elemene by GC-MS, germacrene A is known to undergo a Cope rearrangement to form *β*-elemene under high-temperature conditions (de Kraker et al., 1998). Therefore, the authentic enzymatic product of LcTPS3 is germacrene A rather than *β*-elemene.

### 3.3 *In vitro* activity and kinetics of LcTPSs

The functions of six sesquiterpene synthases were further confirmed by *in vitro* enzymatic assays using purified recombinant protein incubated with GPP, FPP, and GGPP as substrates (**Supplementary Figure S1**). GC-MS analysis demonstrated that all six LcTPSs exclusively utilized FPP, while no detectable products were observed with GPP or GGPP (**Supplementary Figure S2**).

Kinetic parameters were determined using FPP as the substrate (**Table 1**). Among the six enzymes, LcTPS4 exhibited the highest catalytic efficiency (*k*cat/*K*m = 7.06 × 10^4^ M^−1^ s^−1^) and the lowest *K*m value (0.59 μM), indicating strong substrate affinity and efficient catalysis. LcTPS3 and LcTPS7 also showed relatively high catalytic efficiencies (2.26 and 2.10 × 10^4^ M^−1^ s^−1^, respectively). In contrast, LcTPS6 displayed markedly lower catalytic efficiency (0.04 × 10^4^ M^−1^ s^−1^), consistent with its low turnover rate, while LcTPS8 exhibited relatively low catalytic efficiency. LcTPS5 showed moderate catalytic efficiency among the six enzymes.

**Table 1:** Kinetic parameters and fermentation titers of LcTPS3–LcTPS8.

Gene	*K*m [μM]	*k*cat [×10–^2^ s^−1^]	*k*cat/*K*m [× 10^4^ M^−1^s^−1^]	Compound	Shake flask yield (mg L^-1^)
LcTPS3	22.27 ± 2.86	50.34 ± 2.99	2.26	germacrene A	332.53 ± 78.30
LcTPS4	0.59 ± 0.19	4.20 ± 0.21	7.06	(+)-5-*epi*-aristolochene	417.39 ± 6.97
LcTPS5	4.22 ± 1.73	4.13 ± 0.44	0.98	*γ*-selinene	257.41 ± 13.53
LcTPS6	5.48 ± 1.64	0.22 ± 0.02	0.04	germacrene D	26.01 ± 9.06
LcTPS7	1.67 ± 0.50	3.52 ± 0.14	2.10	*α*-*trans*-bergamotene	618.73 ± 14.95
LcTPS8	29.76 ± 9.80	5.59 ± 0.95	0.19	(+)-*β*-himachalene	92.87 ± 13.65

### 3.4 Production of sesquiterpenoids

To establish a metabolic engineering platform for sesquiterpene production, six engineered *E. coli* strains were constructed by expressing *LcTPS* genes in a chassis harboring the pBbA5c plasmid, which contains seven genes of the MVA pathway and an FPP synthase to ensure sufficient precursor supply. To enhance carbon availability, glycerol and sodium pyruvate were supplemented during fermentation. Under shake-flask conditions, all six LcTPSs successfully produced their corresponding sesquiterpenes, which were quantified by GC–MS (**Table 1**).

LcTPS7 achieved the highest production level, reaching 618.73 ± 14.95 mg L^−1^ of *α*-*trans*-bergamotene. LcTPS4, LcTPS3, and LcTPS5 also exhibited relatively high production levels, with titers of 417.39 ± 6.97, 332.53 ± 78.30, and 257.41 ± 13.53 mg L^−1^, respectively. In contrast, LcTPS6 and LcTPS8 produced significantly lower amounts of their respective products, with titers of 26.01 ± 9.06 and 92.87 ± 13.65 mg L^−1^. In microbial production systems lacking host strain optimization or metabolic pathway engineering, sesquiterpene titers typically range within the mg L^−1^ level, with some enzymes achieving or surpassing 100 mg L^−1^ (Rinaldi et al., 2022). A representative example is LcTPS3 from *Liriodendron chinense*, which was reported to yield 151.89 mg L^−1^ of germacrene A in *E. coli.* This titer was subsequently elevated to 14.71 g L^−1^ following metabolic engineering optimization in *Saccharomyces cerevisiae* (Cheng et al., 2025), underscoring the impact of pathway engineering on production yields. Against this backdrop, LcTPS3, LcTPS4, LcTPS5, and LcTPS7 in this study achieved titers exceeding 200 mg L^−1^ without specific host strain optimization, placing them within the higher range reported for unoptimized microbial production systems.

Notably, the production titers of most LcTPS enzymes are generally consistent with their *in vitro* catalytic efficiencies, indicating a strong correlation between enzymatic activity and product formation. However, although LcTPS7 does not exhibit the highest catalytic efficiency *in vitro*, it achieves the highest production titer *in vivo*, suggesting that, in addition to catalytic activity, compatibility within the host system may also influence the final production levels (**Table 1**).

### 3.5 Elucidation of the cyclization routes of LcTPS products

**Figure 5:**
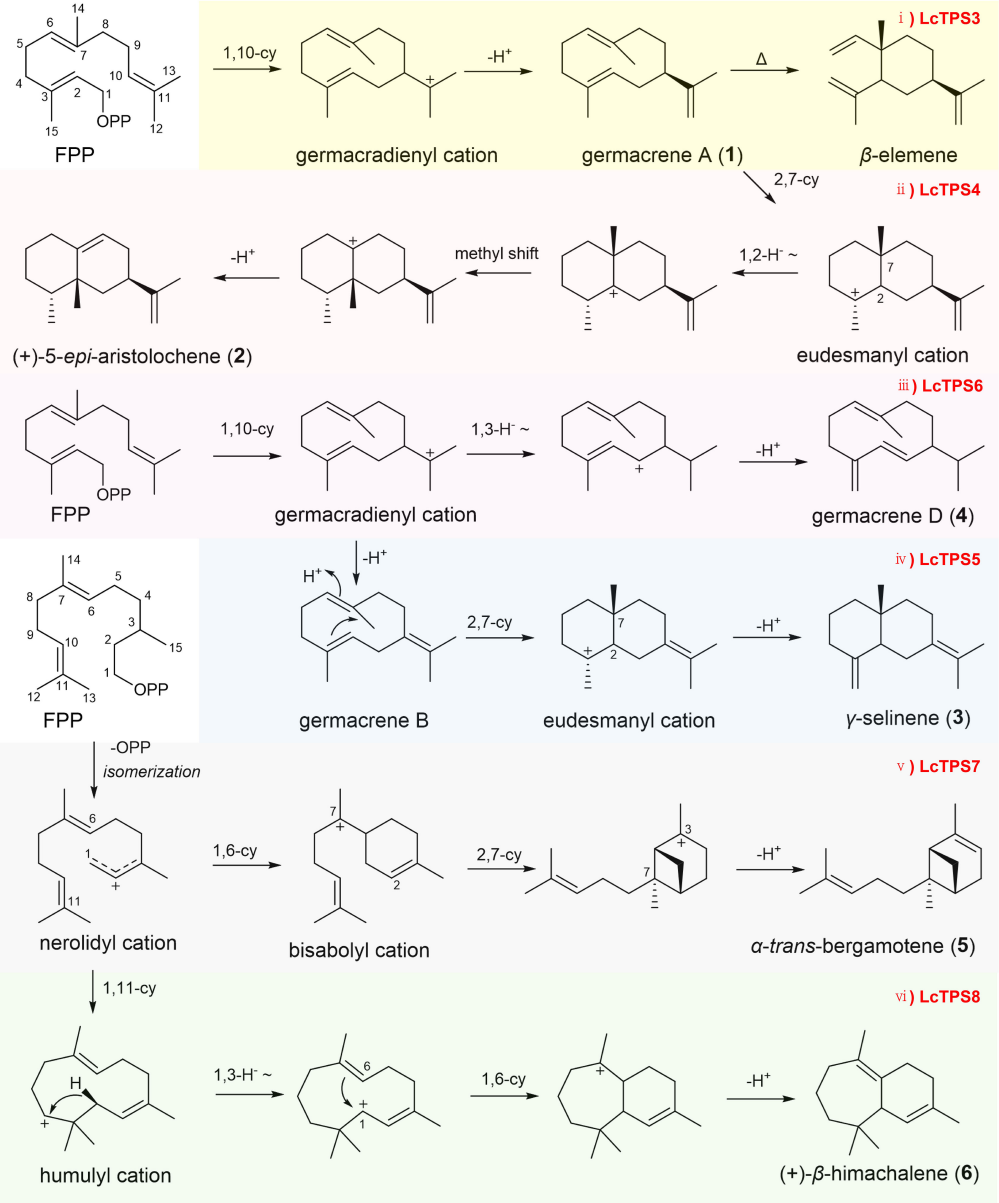
Proposed cyclization routes for products 1–6 of LcTPS3-LcTPS8. i) LcTPS3; ii) LcTPS4; iii) LcTPS6; iv) LcTPS5; v) LcTPS7; vi) LcTPS8.

FPP and its isomer NPP undergo ionization to form abundant carbocation skeletons, which generate diverse sesquiterpenes via deprotonation or sequential cyclization. Based on the catalytic product profiles of the six LcTPSs, their specific cyclization pathways were deduced as follows: LcTPS3, LcTPS4, LcTPS5, and LcTPS6 initially mediate the 1,10-cyclization of FPP to form the germacradienyl cation intermediate. Specifically, LcTPS3 directly catalyzes the deprotonation of the cation to yield germacrene A (**1**) (**Figure 5i**). For LcTPS4, it first catalyzes the conversion of the germacradienyl cation to germacrene A, which subsequently undergoes 2,7-cyclization to generate the eudesmanyl cation. Subsequent 1,2-hydride migration, translocation of the methyl group at the C7 position to the C2 position, and deprotonation yield (+)-5-*epi*-aristolochene (**2**) (**Figure 5ii**). LcTPS6 catalyzes the germacradienyl cation to undergo 1,3-hydride migration before deprotonation, resulting in the formation of germacrene D (**4**) (**Figure 5iii**). LcTPS5 catalyzes the deprotonation of the germacradienyl cation to form germacrene B, which then undergoes 2,7-cyclization to produce γ-selinene (**3**) (**Figure 5iv**).

In contrast, LcTPS7 and LcTPS8 first isomerize FPP to NPP. LcTPS7 mediates 1,6-cyclization of NPP to form the bisabolyl cation. The cation then undergoes 2,7-cyclization and deprotonation to generate *α*-*trans*-bergamotene (**5**) (**Figure 5v**). LcTPS8 catalyzes the 1,11-cyclization of NPP to form the humulyl cation, which undergoes sequential 1,3-hydride migration, 1,6-cyclization, and deprotonation to produce (+)-*β*-himachalene (**6**) (**Figure 5vi**).

### 3.6 Molecular docking elucidates cyclization mechanisms

**Figure 6:**
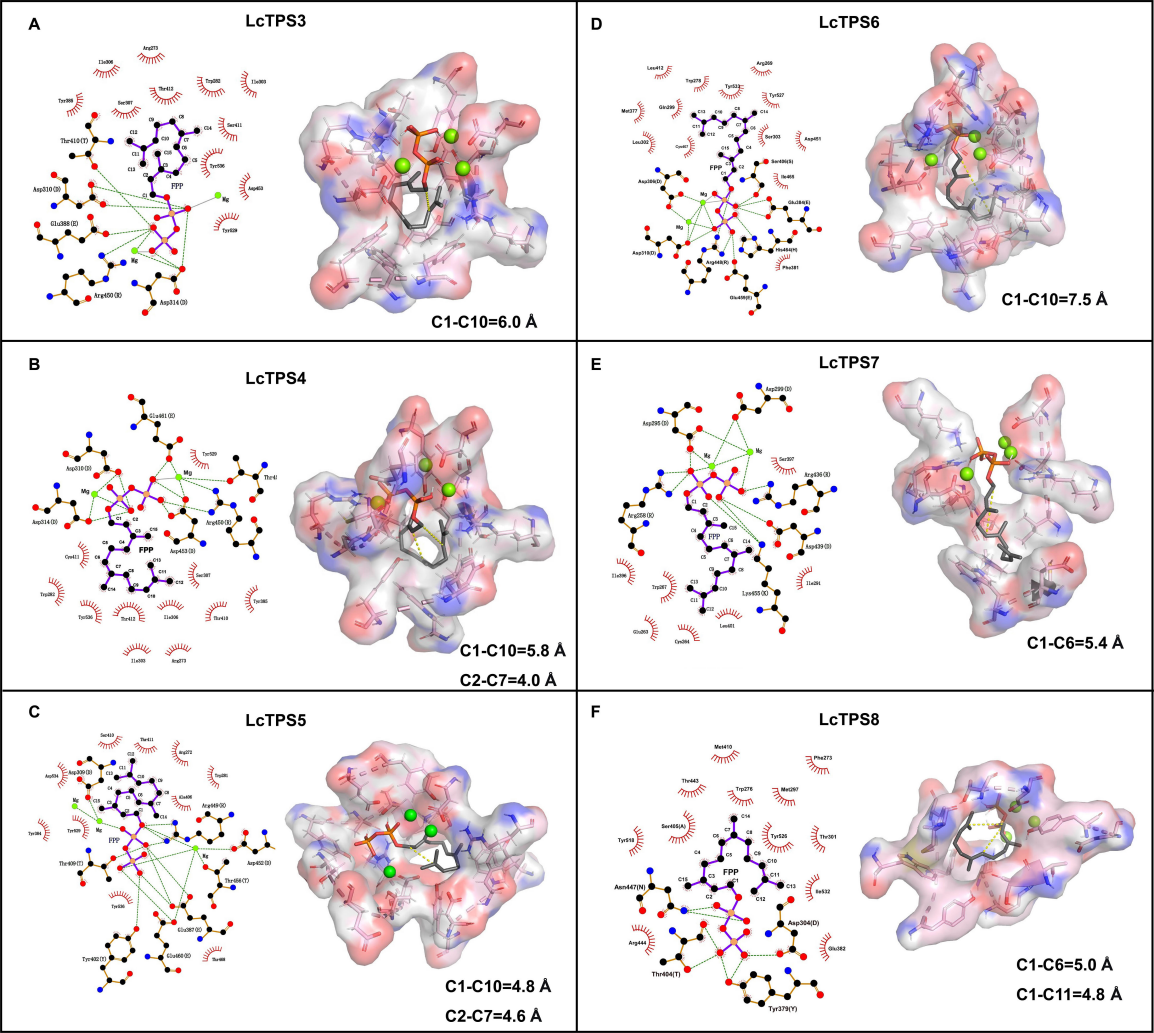
Molecular docking of FPP in the active sites of LcTPS3-LcTPS8. Panels **(A–F)** show 2D interaction diagrams and 3D views of the predicted binding poses of FPP in the catalytic pockets of LcTPS3 **(A)**, LcTPS4 **(B)**, LcTPS5 **(C)**, LcTPS6 **(D)**, LcTPS7 **(E)**, and LcTPS8 **(F)**. Key interacting residues are indicated, and selected interatomic distances (Å) are labeled below each structure.

To elucidate the cyclization mechanism of LcTPSs, their protein structures were predicted using AlphaFold 3. The molecular docking results reveal conformation-specific binding of FPP in LcTPS active pockets, where key residues stabilized FPP via hydrogen bonding and hydrophobic interactions, ensuring that the carbon atoms to be cyclized are in spatially adjacent positions with orientations conducive to bond formation (**Figure 6**). Precise measurements of the distances between carbon atoms at key cyclization sites show significant differences among LcTPS active pockets, further confirming the structural basis for cyclization specificity.

Based on 1,10-cyclization, the distances from C1 to C10 of FPP in LcTPS3 and LcTPS6 are 6.0 Å and 7.5 Å, respectively (**Figures 6A, D**). The shorter C1–C10 distance in LcTPS3 is consistent with its higher catalytic efficiency (*k*cat/*K*m = 2.26 × 10^4^ M^−1^ s^−1^) compared to LcTPS6 (0.04 × 10^4^ M^−1^ s^−1^), which is further reflected in its approximately 12-fold higher product titer. Both LcTPS4 and LcTPS5 undergo 2,7-cyclization to form eudesmanyl cation; the C2-C7 distances of FPP in them are 4.0 Å and 4.6 Å, respectively (**Figures 6B, C**). The shorter distance in LcTPS4 may facilitate the methyl migration from C7 to C2, which is consistent with its approximately 7-fold higher catalytic efficiency compared to LcTPS5 (**Figure 5ii**, **Table 1**). In addition, FPP has shown the embryonic form of bisabolyl cation configuration in the active pocket of LcTPS7 (**Figure 6E**).

Structurally, (+)-*β*-himachalene could form via 1,6-cyclization followed by 1,11-cyclization. However, molecular docking of LcTPS8 with FPP showed C1-C11 distance (4.8 Å) shorter than C1-C6 (5.0 Å) (**Figure 6F**), indicating preference for initial 1,11-cyclization. This finding is consistent with the reported mechanism of a multiproduct *β*-himachalene synthase in *Cryptosporangium arvum*, where initial 1,11-cyclization was deduced from by-products (Rinkel and Dickschat, 2019).

### 3.7 Expression patterns of *LcTPSs*

**Figure 7:**
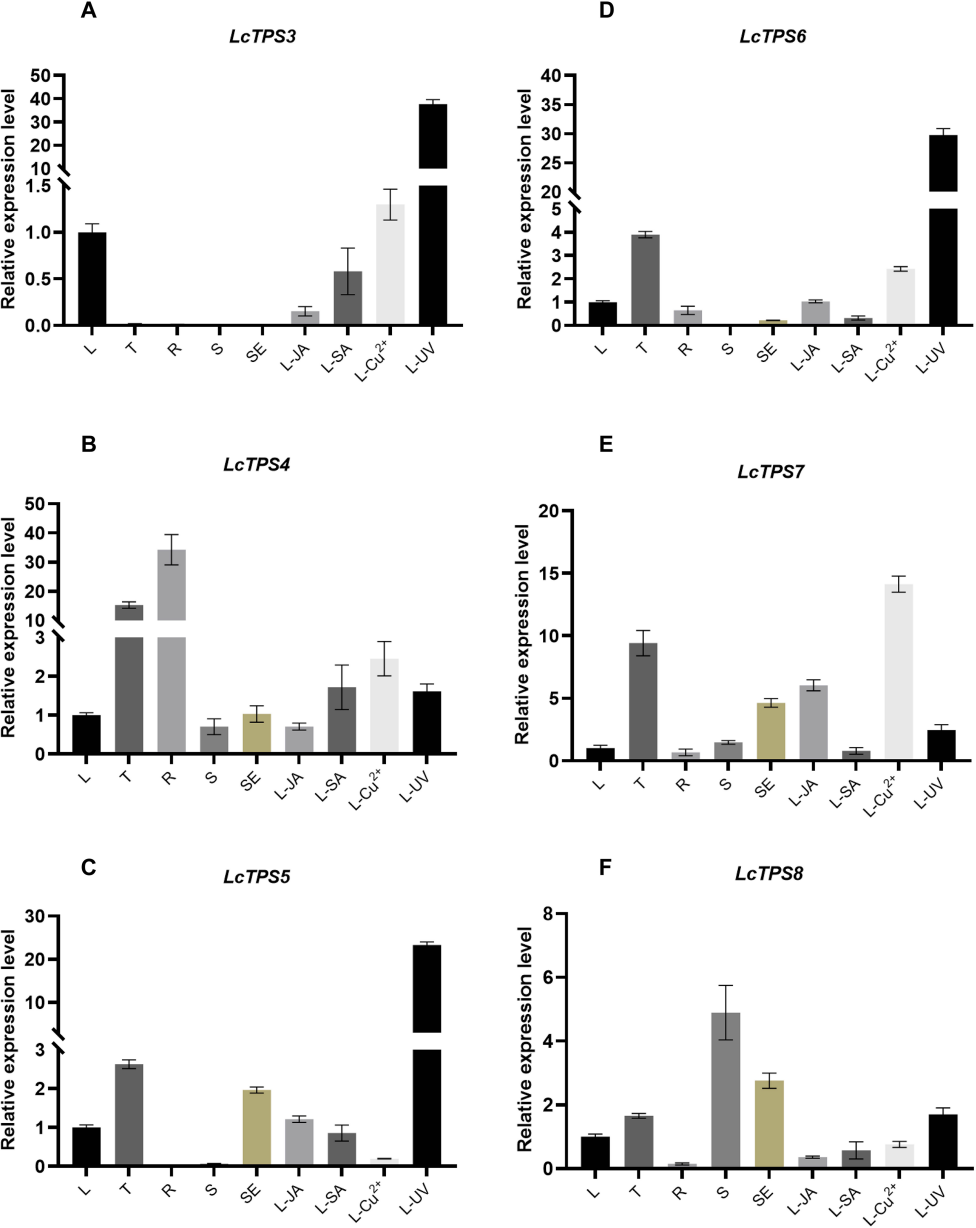
Expression patterns of *LcTPSs* in *L. canum*. **(A–F)** Expression patterns of *LcTPS3*-*LcTPS8* across different tissues and under various stress conditions. L, leaf; T, trichome; SE, stem epidermis; S, epidermis-free stem; R, root; JA, methyl jasmonate-treated leaves; SA, salicylic acid-treated leaves; Cu^2+^, CuCl_2_-treated leaves; UV, UV-irradiated leaves.

The expression patterns of the six TPS genes were analyzed in different tissues of *L. canum* and under various stress conditions. All six TPS genes were expressed in leaves, yet each displayed distinct tissue-specific and stress-responsive expression characteristics (**Figure 7**). *LcTPS3* was specifically highly expressed in leaves and was upregulated under UV stress treatment (**Figure 7A**). *LcTPS4* showed high expression levels in trichomes and roots but was unresponsive to any of the tested stress treatments (**Figure 7B**). *LcTPS5* was highly expressed in trichomes (T) and stem epidermis (SE) and was significantly upregulated by UV stress (**Figure 7C**). *LcTPS6* was highly expressed in trichomes and significantly induced by UV stress (**Figure 7D**). *LcTPS7* exhibited high expression levels in trichomes (T) and stem epidermis (SE), and its expression was induced by treatments with jasmonic acid (JA) and copper chloride (CuCl_2_). This suggests a potential role of *LcTPS7* and its product in the biotic and abiotic stress defense responses of *L. canum* (**Figure 7E**). In contrast, *LcTPS8* showed high expression in stems but did not exhibit stress-induced expression (**Figure 7F**).

## 4 Discussion

Sesquiterpenoids are important plant secondary metabolites with diverse biological activities and key roles in defense against biotic and abiotic stresses (Fraga, 2012). Furthermore, they are also indispensable resources in pharmaceutical research and production (Jiang et al., 2021; Wang et al., 2023; Xu et al., 2025). This study systematically characterized six functionally distinct sesquiterpene synthases (LcTPS3–LcTPS8) from *L. canum*. Heterologous expression in *E. coli* combined with product isolation and structural identification revealed their distinct catalytic activities, producing germacrene A (**1**), (+)-5-*epi*-aristolochene (**2**), *γ*-selinene (**3**), germacrene D (**4**), *α*-*trans*-bergamotene (**5**), and (+)-*β*-himachalene (**6**), respectively.

The notable structural differences among these six products highlight the functional diversity within the sesquiTPS family in *L. canum*. qRT-PCR analysis indicated tissue-specific expression and differential stress responses, suggesting a spatially organized terpenoid metabolic network. Specifically, the UV-responsive induction of *LcTPS3*, *LcTPS5*, and *LcTPS6*, which produce germacrene A, γ-selinene, and germacrene D, respectively, suggests that their products may play a role in antioxidant biosynthesis, potentially mitigating UV-induced damage. Additionally, *LcTPS4*, *LcTPS5*, *LcTPS6*, and *LcTPS7* displayed high expression levels in trichomes, implying that (+)-5-*epi*-aristolochene, *γ*-selinene, germacrene D, and *α*-*trans*-bergamotene may play a role in herbivore defense. Furthermore, LcTPS4 was also highly expressed in roots, where its product may be involved in root allelopathy and the regulation of rhizosphere microbial communities. This function is analogous to that of (+)-5-*epi*-aristolochene synthase (5EAS) in *Nicotiana tabacum*, which synthesizes the precursor of the phytoalexin capsidiol, thereby conferring resistance against the pathogen *Phytophthora infestans* (Yin et al., 1997).

Distinct cyclization modes of the six LcTPSs were inferred from enzymatic product profiles and molecular docking, providing mechanistic insights into terpene synthase specificity relevant for enzyme-guided bioproduction (Degenhardt et al., 2009; Hong and Tantillo, 2014; Christianson, 2017). The core mechanistic difference lies in the cyclization initiation sites on the FPP carbon skeleton, giving rise to four canonical modes: 1) 1,10-cyclization mediated by LcTPS3 and LcTPS6, producing germacrene-type monocyclic sesquiterpenes; 2) a 1,10- and 2,7-cyclization cascade by LcTPS4 and LcTPS5, forming bicyclic sesquiterpenes; 3) 1,6-cyclization by LcTPS7, yielding bisabolene-type sesquiterpenes; and 4) 1,11-cyclization by LcTPS8, generating humulene-type sesquiterpenes. Molecular docking revealed that the spatial conformation of FPP within the active sites of LcTPSs is closely aligned with the resulting cyclization products, thereby influencing the cyclization initiation site and reaction trajectory. Notably, distinct cyclization modes may affect the formation and stability of carbocation intermediates, thereby modulating catalytic efficiency and contributing to the observed differences in kinetic parameters and product titers among LcTPSs. Our results offer important insights for a deeper understanding of the structure-function relationship of terpene synthases. With the rapid advancement of artificial intelligence, sequence- and structure-based deep learning predictions have become increasingly accurate, providing powerful tools for elucidating enzyme catalytic mechanisms and significantly accelerating research into natural product biosynthetic pathways.

Using metabolic engineering, we constructed engineered *E. coli* strains capable of producing six sesquiterpenoids, with LcTPS3, LcTPS4, LcTPS5, and LcTPS7 strains reaching yields of 257.41–618.73 mg L^−1^ in shake-flask cultures (**Table 1**). These results demonstrate that LcTPSs can serve as efficient biocatalysts for sesquiterpenoid production in microbial systems. Several of the identified products show potential for application. Germacrene A, produced by LcTPS3, is a key precursor of *β*-elemene, a clinically used antitumor agent, and its microbial production provides an alternative to plant extraction (Dai et al., 2025; Liu et al., 2026). In addition, (+)-5-*epi*-aristolochene and *α*-*trans*-bergamotene, together with their derivatives, have been reported to exhibit anti-inflammatory and antifungal activities (Dhama et al., 2023; Veiga Junior et al., 2007; Zhao et al., 2004). *γ*-selinene has also been proposed as a precursor of dihydroagarofuran-type sesquiterpene alkaloids with pesticidal and piscicidal activities (Liu et al., 2025), and its production in this study provides a basis for exploring potential agricultural applications. Collectively, these products highlight the potential of LcTPSs as versatile enzymatic resources for the biosynthesis of structurally diverse and functionally relevant terpenoids. Future studies may further exploit these enzymes through synthetic biology strategies, including site-directed mutagenesis and structure-guided engineering, to enhance product yield. Overall, these findings advance our understanding of the terpenoid metabolic network in *L. canum* and provide valuable enzymatic resources for sustainable sesquiterpenoid biosynthesis.

## Data Availability

The datasets presented in this study can be found in online repositories. The names of the repository/repositories and accession number(s) can be found in the article/**Supplementary Material**.
