# Supplementary material for: Integrated characterization of six sesquiterpene synthases unravels diversified terpene biosynthesis in the mint species Leucosceptrum canum
Source: Front Plant Sci. 2026 Apr 28;17:1833733. doi: 10.3389/fpls.2026.1833733 (PMC13162333; doi:10.3389/fpls.2026.1833733)
Supplement: Supplementary file 1 [file DataSheet1.docx]

Supplementary Material

**Contents**

**Supplementary Figures:**

**Table S1**. Sequence parameters and functional predictions of LcTPS3–LcTPS8

**Table S2**. Terpene synthases used in phylogenetic tree construction.

**Table S3**. Plasmids used in this study.

**Table S4**. Primers used in this study.

**Figure S1.** SDS-PAGE analysis of purified LcTPS proteins.

**Figure S2.** *In vitro* enzymatic characterization of LcTPSs.

**Figure S3-12.** NMR spectra of compounds **1-4, 6**

**Table S1**. Sequence parameters and functional predictions of LcTPS3–LcTPS8

| **Gene name** | **Length /bp** | **Prediction Function** | **Molecular Weight /kDa** | **Theoretical pI** |
| --- | --- | --- | --- | --- |
| LcTPS3 | 1674 | 5-*epi*-aristolochene | 65.03 | 5.1 |
| LcTPS4 | 1674 | Germacrene A synthase | 64.93 | 5.05 |
| LcTPS5 | 1674 | Germacrene A synthase | 64.76 | 5.18 |
| LcTPS6 | 1662 | Germacrene D synthase | 65.18 | 5.25 |
| LcTPS7 | 1629 | *α*-*trans*-bergamotene synthase | 63.06 | 5.48 |
| LcTPS8 | 1662 | *γ*-cadinene synthase | 64.65 | 5.02 |

**Table S2**. Terpene synthases used in phylogenetic tree construction.

| Subfamily | Name | Function | Accession number | Species |
| --- | --- | --- | --- | --- |
| TPS-a | LpGerAS | [germacrene A synthase](https://pubchem.ncbi.nlm.nih.gov/compound/9548706) | AGN72800.1 | *Lavandula pedunculata* |
|  | LaGerDS | germacrene D synthase | AAR99061.1 | *Populus trichocarpa x Populus deltoides* |
|  | SlGerCS | germacrene C synthase | AAC39432.1 | *Solanum lycopersicum* |
|  | ObGerDS | germacrene D synthase | AAV63786.1 | *Ocimum basilicum* |
|  | TEAS | 5-*epi*-aristolochene | Q40577.3 | *Nicotiana tabacum* |
|  | PcPS | patchoulol synthase | AAS86323.1 | *Pogostemon cablin* |
|  | LaCarS | *β*-caryophyllene synthase | AGL98419.1 | *Lavandula angustifolia* |
|  | OvCarS | (*E*)-*β*-caryophyllene synthase | ADK73616.1 | *Origanum vulgare* |
|  | ObTPS1 | (*E*)-*β*-caryophyllene | AIJ00878.1 | *Oryza barthii* |
|  | HISTS1 | Alpha-humulene synthase | ACI32639.1 | *Humulus lupulus* |
|  | PcCurS | *γ*-curcumene synthase | AAS86319.1 | *Pogostemon cablin* |
|  | Lc-CedS | cedrol synthase | QBP05430.1 | *Leucosceptrum canum* |
|  | RcSeTPS1 | [(-)-alpha-copaene](https://pubchem.ncbi.nlm.nih.gov/compound/442355) | AEQ27766.1 | *Ricinus communis* |
|  | ObCadS | *γ*-cadinene synthase | AAV63787.1 | *Ocimum basilicum* |
|  | LaCADS | *γ*-Cadinene /*τ*-Cadinol synthase | AGL98418.1 | *Lavandula angustifolia* |
|  | ObSES | selinene synthase | AY693643 | *Ocimum basilicum* |
|  | CiCopS | *β*-copaene synthase | WIM34773.1 | *Chrysanthemum indicum* |
|  | LcTPS2 | di-/sesterTPS | MZ147599.1 | *Leucosceptrum canum* |
| TPS-b | CcTPS1 | Mono-/sesqui-/di-/sesterTPS | QZL13763.1 | *Colquhounia coccinea var. mollis* |
|  | CcTPS2 | (*R*)-*β*-bisabolene | OQ551732.1 | *Colquhounia coccinea var. mollis* |
|  | ZaFarS | *α*-farnesene synthase | AYQ58361.1 | *Zanthoxylum ailanthoides* |
|  | CsFarS | *α*-farnesene synthase | UFQ06454.1 | *Camellia sinensis* |
|  | SaSS | santalene synthase | AGV01243.1 | *Santalum album* |
|  | SaBS | bisabolene synthase | AIV42941.1 | *Santalum album* |
|  | VvLinS | MonoTPS | ADR74209.1 | *Vitis vinifera* |
|  | SfCinS1 | MonoTPS | ABH07677.1 | *Salvia fruticosa* |
| TPS-c | GrLPPS | labda-13-en-8-ol diphosphate synthase | AGN70887.1 | *Grindelia robusta* |
|  | SmCPS2 | *nor*-copalyl diphosphate synthase | AHJ59322.1 | *Salvia miltiorrhiza* |
|  | CfTPS2 | 8*α*-hydroxy-CPP synthase | KF444507.1 | *Coleus forskohlii* |
|  | OsCPS4 | *syn*-copalyl diphosphate synthase | NP_001389265.1 | *Oryza sativa* |
|  | OsCPS2 | *ent*-copalyl diphosphate synthase | NP_001403441.1 | *Oryza sativa* |
| TPS-d | AgSelS | *δ*-selinene synthase | AAC05727.1 | *Abies grandis* |
|  | PaLonS | longifolene synthase | AAS47695.1 | *Picea abies* |
|  | AgTerS | MonoTPS | AAF61454.1 | *Abies grandis* |
|  | PsTPS3 | longifolene synthase | ABV44454.1 | *Pinus sylvestris* |
|  | CnVS | valencene synthase | AFN21429.1 | *Callitropsis nootkatensis* |
| TPS-e/f | SmKSL1 | miltiradiene synthase | ABV08817.1 | *Salvia miltiorrhiza* |
|  | CfTPS4 | miltiradiene / manoyl oxide synthase | KF444509.1 | *Coleus forskohlii* |
|  | SsSS | sclareol synthase | AET21246.1 | *Salvia sclarea* |
|  | OsKSL7 | *ent*-cassa-12,15-diene synthase | BAC56714.1 | *Oryza sativa* |
| TPS-g | AmMyrS | Mono-/sesqui-TPS | AY195608.1 | *Antirrhinum majus* |
|  | ApLinS | MonoTPS | ADD81295.1 | *Actinidia polygama* |
|  | AmLins | MonoTPS | EF433761.1 | *Antirrhinum majus* |
|  | AtTPS14 | MonoTPS | NP_176361.2 | *Arabidopsis thaliana* |

**Table S3.** Plasmids used in this study.

| Plasmids | Description | Purpose | Source |
| --- | --- | --- | --- |
| pET28-MmGFDPS | GFDPS (Geranylfarnesyl diphosphate synthase from the archaeon *Methanosarcina mazei*) is cloned into the *BamH* I/ *Xho* I sites of pET28 | produce GFPP, GGPP, FPP, and GPP | This lab collection |
| pBbA5c | acetoacetyl-CoA synthase gene, IDP isomerase gene, and FDP synthase gene from *E. coli*, and HMGCoA synthase gene, a truncated version of HMGCoA reductase gene, mevalonate kinase gene, phosphomevalonate kinase gene, and phosphomevalonate decarboxylase gene from Saccharomyces cerevisiae, with codon optimization | produce FPP | This lab collection |
| pET32-LcTPS3 | LcTPS3 sequence is cloned into the *BamH* I/ *Xho* I sites of pET32a | functional research | This study |
| pET32-LcTPS4 | LcTPS4 sequence is cloned into the *BamH* I/ *Xho* I sites of pET32a | functional research | This study |
| pET32-LcTPS5 | LcTPS5 sequence is cloned into the *BamH* I/ *Xho* I sites of pET32a | functional research | This study |
| pET32-LcTPS6 | LcTPS6 sequence is cloned into the *BamH* I/ *Xho* I sites of pET32a | functional research | This study |
| pET32-LcTPS7 | LcTPS7 sequence is cloned into the *BamH* I/ *Xho* I sites of pET32a | functional research | This study |
| pET32-LcTPS8 | LcTPS8 sequence is cloned into the *BamH* I/ *Xho* I sites of pET32a | functional research | This study |

**Table S4**. Primers used in this study.

| Primer name | Sequence (5’→3’) |
| --- | --- |
| For heterologous expression in *E. coli* | |
| *LcTPS3*-p32-F | gccatggctgatatcggatccATGGACTCCAAAGAAATCAAGAATAA |
| *LcTPS3*-p32-R | gtggtggtggtggtgctcgagTCAAATGTCGAAGGAATCGAGG |
| *LcTPS4*-p32-F | gccatggctgatatcggatccATGGATCCAGCGAATGCCGATGTTGC |
| *LcTPS4*-p32-R | gtggtggtggtggtgctcgagCTATATCTTAATTTGATCGACGAACAAA |
| *LcTPS5*-p32-F | gccatggctgatatcggatccATGGCTCCAAAGAATGCAGATAT |
| *LcTPS5*-p32-R | gtggtggtggtggtgctcgagCTAAATCTTGATTTGATCAACGAACAAA |
| *LcTPS6*-p32-F | gccatggctgatatcggatccATGGATTTGAAAAACCAAACTGTTG |
| *LcTPS6*-p32-R | gtggtggtggtggtgctcgagTCATGGAAGAGGGTCAATGTAGAG |
| *LcTPS7*-p32-F | gccatggctgatatcggatccATGACGGAGGCAAGGAGATCA |
| *LcTPS7*-p32-R | gtggtggtggtggtgctcgagTCATGACATAGGGATGGGTTCA |
| *LcTPS8*-p32-F | gccatggctgatatcggatccATGAGTGATGCTAATGTATGCCTCC |
| *LcTPS8*-p32-R | gtggtggtggtggtgctcgagTTAGAGGAGTATGGGATCCACAAGT |
| qRT-PCR primers for gene expression | |
| *LcTPS3*-q-F | TGTGGAGGCCAAGTGGTT |
| *LcTPS3*-q-R | ATGTGGTCGCGAGCAAGT |
| *LcTPS4*-q-F | ACGCAACACACTACGCACTA |
| *LcTPS4*-q-R | ATTCCTCGAAGGGTGGCAAG |
| *LcTPS5*-q-F | AACAAGTTGCGCATGCCC |
| *LcTPS5-*q-R | TGGCAAACCTCAGGAGCG |
| *LcTPS6*-q-F | TTAAGCAATCCTGTCGCGGA |
| *LcTPS6*-q-R | GCTTCGACACGCGTTAATCC |
| *LcTPS7*-q-F | TCGTTGGCCTTCCATGGT |
| *LcTPS7*-q-R | AGAACACCCTGTGTCGCG |
| *LcTPS8*-q-F | TAGCTACCTTCCCTGGCATG |
| *LcTPS8*-q-R | ACCATTTCTCCTCCTCTGCT |
| *LcActin*-qRT-F | GAGCATCCGATTCTCTTGACTG |
| *LcActin*-qRT-R | ATAGATGGGGACTGTATGGCTG |

**
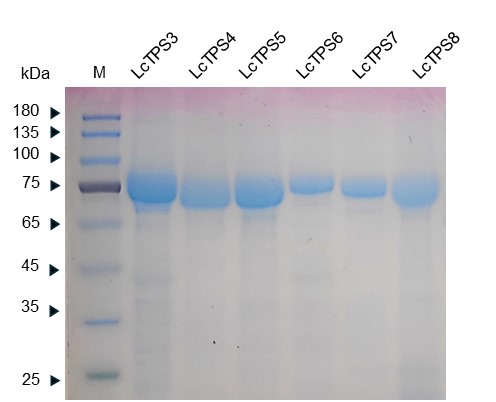
**

**Figure S1. SDS-PAGE analysis of purified LcTPS proteins.** Purified recombinant LcTPS3, LcTPS4, LcTPS5, LcTPS6, LcTPS7, and LcTPS8 were analyzed by SDS-PAGE. M, protein molecular weight marker. The predicted molecular weights of these proteins are listed in Table S1.

**
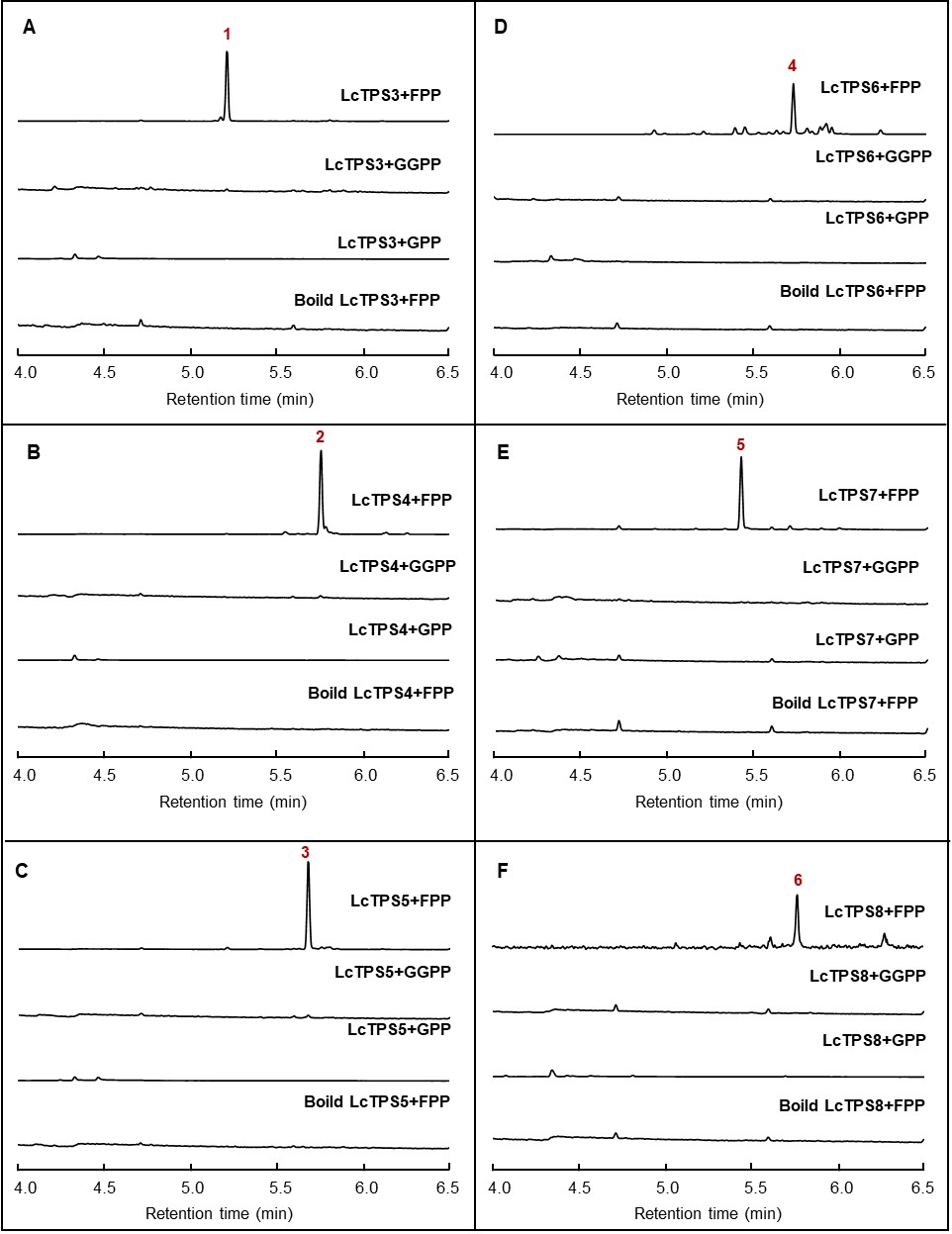
**

**Figure S2. *In vitro* enzymatic characterization of LcTPSs.** (**A**–**F**) GC-MS analysis of the reaction products generated by purified LcTPS3, LcTPS4, LcTPS5, LcTPS6, LcTPS7, and LcTPS8, respectively, using GPP, FPP, or GGPP (50 μM) as substrates. Boiled (heat-inactivated) enzymes were included as negative controls. Peaks labeled **1**–**6** correspond to the products generated by LcTPSs using FPP as the substrate.

**Figure S3**. ^1^H NMR spectrum of (**1**) in C_6_D_6_ (600 MHz)

 **Figure S4**. ^13^C NMR spectrum of (**1**) in C_6_D_6_ (150 MHz)

**Figure S5**. ^1^H NMR spectrum of (**2**) in CDCl_3_ (600 MHz)

**Figure S6.** ^13^C NMR spectrum of (**2**) in CDCl_3_ (150 MHz)

**Figure S7.** ^1^H NMR spectrum of (**3**) in CDCl_3_ (600 MHz)

**Figure S8.** ^13^C NMR spectrum of (**3**) in CDCl_3_ (150 MHz)

**Figure S9**. ^1^H NMR spectrum of (**4**) in CDCl_3_ (600 MHz)

**Figure S10**. ^13^C NMR spectrum of (**4**) in CDCl_3_ (150 MHz)

**Figure S11**. ^1^H NMR spectrum of (**6**) in CDCl_3_ (600 MHz)

**Figure S12**. ^13^C NMR spectrum of (**6**) in CDCl_3_ (150 MHz)
